# Change in Electrical Resistance of SMA (NiTi) Wires during Cyclic Stretching

**DOI:** 10.3390/s22093584

**Published:** 2022-05-08

**Authors:** Sebastian Sławski, Marek Kciuk, Wojciech Klein

**Affiliations:** 1 Department of Theoretical and Applied Mechanics, Silesian University of Technology, Konarskiego 18A, 44-100 Gliwice, Poland; wojciech.klein@polsl.pl; 2 Department of Mechatronics, Silesian University of Technology, Akademicka 2A, 44-100 Gliwice, Poland; marek.kciuk@polsl.pl

**Keywords:** NiTi, self-sensing, health monitoring, resistance, sensor, stretching, cyclic testing, martensite reorientation

## Abstract

In this article, the use of Nickel Titanium (NiTi) alloy as a sensor is examined. A cyclic stretching test, that has various elongations (0.5 and 1%), is administered to NiTi wires with various diameters and lengths. It is assumed that the elongation enables an observation of the change in electrical resistance of the NiTi wires, due to martensite reorientation. During the test, the stretching force, the displacement, and the electrical resistance of the NiTi wires are measured. Following the test, the resistance of all the tested samples differed compared to the starting position. Conducted research indicates that NiTi wires are very sensitive to resistance change when they are deformed. A large difference in NiTi electrical resistance was visible in all samples during the first stretching cycle. For longer wires, with a smaller diameter, resistance change was visible during almost all of the stretching cycles. However, the observed changes were very small. Based on the obtained results, it can be justifiably stated that NiTi wires could be used to build deformation sensors, which operate both online and offline. Moreover, NiTi wires with a small diameter could be used to create cyclic loading sensors. Such sensors can be used in self-sensing applications or in structural health monitoring.

## 1. Introduction

Nowadays, the development of technology continues to be extremely fast. Novel designs, developed by scientists and engineers, are often very complex [1,2,3,4]. All newly designed devices require a lot of actuators and sensors, so that they can operate as intended. Various types of sensors are used to measure the influence of external forces and working conditions [5,6,7,8], alongside prediction of material properties when tested in laboratories [9,10,11,12]. Therefore, there is a need to find newer solutions to enable the development of novel measurement methods. An interesting solution is to build new devices and measuring systems with smart materials. These materials are defined as substances that have one or more properties that can be significantly modified in a controlled fashion by external stimuli, such as stress, moisture, electric or magnetic fields, light, temperature, pH, or chemical compounds [13]. Smart materials are increasingly used in new designs that operate based on, for example, piezoelectric effects (such as a griper [14]) or magnetic fluids (a suspension system [15] and a clutch [16]). 

Shape memory alloys (SMAs) belong to a group of smart materials that change their shape and material properties under the influence of external factors, such as, for example, heat or mechanical deformation [17,18]. SMAs were discovered in the 1930s, but their first applications arose in the 1960s when Nitinol (nickel and titanium (NiTi) alloy) was invented in the Nickel Titanium Naval Ordnance Laboratory. Three structure types for NiTi alloys can be observed for various thermal and mechanical loading combinations. The basic structure (in which no external source of heat or mechanical load is supplied) is known as the martensite phase. This phase encompasses both the twinned and de-twinned martensite phases. Detwinned martensite is formed during the mechanical material deformation of the twinned martensite, in a process called martensite reorientation. Any changes caused by this transformation are permanent at low temperatures (i.e., those below the temperature that initiates the austenite phase transformation). However, the changes could be restored by temporarily modifying the martensite phase to the austenite one; this transformation is related to a shape changing mechanism and it is controlled by temperature. NiTi alloys can be divided into the categories of low temperature (LT) and high temperature (HT), which depends on the temperature that initiates the transformation between the martensite and the austenite phases. During this heat-based transformation, the shape, and some of the properties, of the materials are changed (e.g., Young modulus and electrical resistance). When the temperature of NiTi wire falls below the transformation phase temperature (i.e., from austenite to martensite), the material returns to the martensite phase. During this transformation, all the properties of the material are restored to the values that correspond to the martensite phase [17,19,20]. Due to the unique properties of the SMA, many applications are possible throughout a wide range of fields, which includes civil engineering, space and aircraft science, and medicine [21,22,23,24,25,26,27,28,29].

All the mentioned phase transformations of NiTi wires have an impact on their electrical resistance. The latter is complex since it depends on many factors, such as temperature, r-phase distortion, stress, deformation, phase transformation, and martensite reorientation [18]. Thus, NiTi sensors can be based on thermally or mechanically induced resistance changes. Liu et al. [30] state that SMAs are strong candidates for sensor applications because of their sensitivity. Rączka et al. [31] reported that NiTi resistance measurements could be used as feedback variables that enable positioning of systems based on thin NiTi wires. Šittner et al. [32] measured change in electrical resistance of NiTi wires, which allowed an investigation into the thermomechanical behavior of the R-phase. During an analysis on the electrical resistivity of NiTi during thermal cycles and under constant tensile stress, Uchil et al. [33] found that electrical resistivity is dependent on the number of performed cycles. Wu et al. [34] examined the change in electrical resistance of NiTi wire and stated that the stress-induced martensite reorientation has little influence on the slope of the curves. However, they also noticed that the relationship between stress and electrical resistance of NiTi wire is complicated. Moreover, the electrical resistivity of NiTi during heating and cooling stages has been investigated by Antonucci et al. [35]. The results they obtained showed that the NiTi phase can be identified from electrical resistivity measurements. The electrical resistance of NiTi wires, when acted upon by thermal and mechanical effects, has been investigated by Novák et al. [36]. They discovered that the electrical resistivity of the martensite phase greatly increases when martensite reorientation is under tension.

Electrical resistance measurements of NiTi wires can also be used as feedback in self-sensing applications. The linearity and repeatability of the stress-resistance curve [37], determined by Furs et al. makes these materials ideal for applications that use resistance measurements as a position feedback sensor. NiTi wires are also used by Li et al. [38], who proposed a new repair method for simple RC beams strengthened with carbon fiber reinforced polymers. In this method, Li et al. used the relationship between the deflection and the change in rate of electrical resistance of NiTi wires. This correlation provides a potentially useful tool for developing smart RC structures with integrated self-diagnosis and self-repairing functionality. Wang et al. [39] enabled accurate control of an actuator, which consisted of a pair of NiTi wires, by self-sensing feedback with hysteresis compensation. The development of a fused deposition modeling (FDM) technique increased the possibility of the applications of NiTi wires in polymer-based composite materials, which had been previously presented by Dudek et al. [40].

In this paper, change in the electrical resistance of NiTi wires during cyclic stretching with small elongations is investigated. In the literature, there is currently a lack of research on this topic, which focuses on the electrical resistance change in NiTi wires caused by martensite reorientation that is induced by cyclic stretching with small elongations. The measured change in the electrical resistance can be used to assess the deformation of the structure, or count the number of deformation cycles. Thus, it can be used in self-sensing application and in structural health monitoring.

## 2. Materials and Methods

The research presented concerns cyclic stretching of NiTi wires with small elongations. The performed research assessed the use of NiTi wires in a sensor that operates based on martensite reorientation, which occurs during the mechanical deformation of the material; it is permanent until the NiTi wire is not associated with the martensite-austenite-martensite phase transformation. Therefore, the studied phenomenon can be used to create a NiTi wire-based sensor that can operate both online and offline. Thereby, it was important to assess the influence of the diameter of the NiTi wire on the resistance change during cyclic stretching. Thus, NiTi LT wires of various diameters (i.e., 100, 150, 200, and 250 µm) were employed. Depending on the actual application of the NiTi wires, the length could also be modified. Due to this, NiTi wires with various free lengths (the NiTi wire was placed between fasteners, which locked it into place) were investigated (ca. 150 and 200 mm). During the tests, all the samples underwent cyclic stretching with small elongations (0.5 and 1%). Information about the tested NiTi wire samples is presented in Table 1.

**Figure 1:**
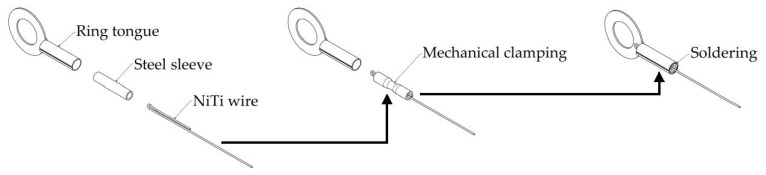
Stages for the creation of the NiTi wire fastening.

Before beginning the test, the NiTi wires were fastened between clamps. To avoid problems with NiTi soldering, a special fastening system was used. The various creation stages for the NiTi wire fastening are presented in Figure 1. For the first stage, the NiTi wire was bent (about 180°). After that, the ends of the bent NiTi wire were positioned inside the steel sleeve. Subsequently, the steel sleeve with the NiTi wire inside it was mechanically clamped (a few times to secure the mechanical connection). Following mechanical clamping, the steel sleeve was positioned inside the ring tongue. The connection between the steel sleeve and the ring tongue was created by soldering. In this case, the free length of the NiTi wire was measured between the ring tongues that were fastened at both ends of the NiTi wire. 

**Figure 2:**
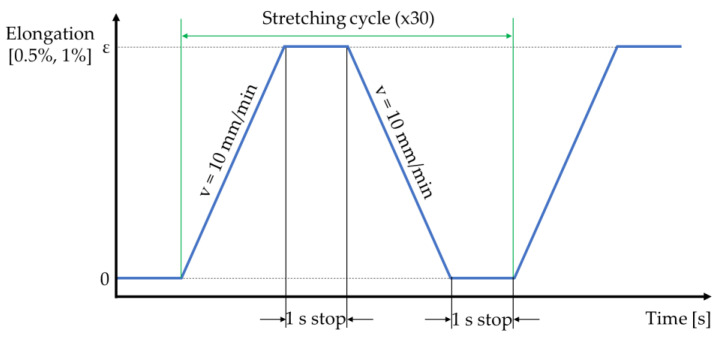
Stretching cycle of NiTi wire.

As previously mentioned, all the prepared NiTi samples were applied to the cyclic stretching test with small elongations (0.5 and 1%). A representation of the stretching cycle is provided in Figure 2. Each stretching cycle began in position “0”, which is when the NiTi wire deformation corresponds to the applied preload (ca. 0.3 N). These preloads were all similar for every analyzed case and the stretching cycle consisted of four stages. In the first stage, the NiTi wire sample was stretched to the required elongation length. The movement speed during this stage was set as 10 mm/min. In the second stage (where the sample had elongation), the movement was stopped for 1s. During the third stage, the wire was clamped back into its initial position “0” (which was set up based on the applied preload); the movement speed for this stage was identical to the first stage (i.e., 10 mm/min). In the fourth stage, the movement again stopped (but in the “0” position) for 1s. The described stretching cycle was repeated 30 times for each test sample. The test conditions assumed that NiTi wires deformed in the martensite phase, which indicates martensite reorientation from the twinned to the detwinned configuration. Martensite reorientation is associated with a change in some of the material properties, such as electrical resistance, although the observed changes in the materials are temporary. Modifications in the material properties can be measured until the NiTi wires do not perform the martensite—austenite—martensite phase transformation, which is related to the application of heat. If the tested samples aligned with the martensite—austenite—martensite phase transformation, their properties would return to initial values (i.e., those before the test).

**Figure 3:**
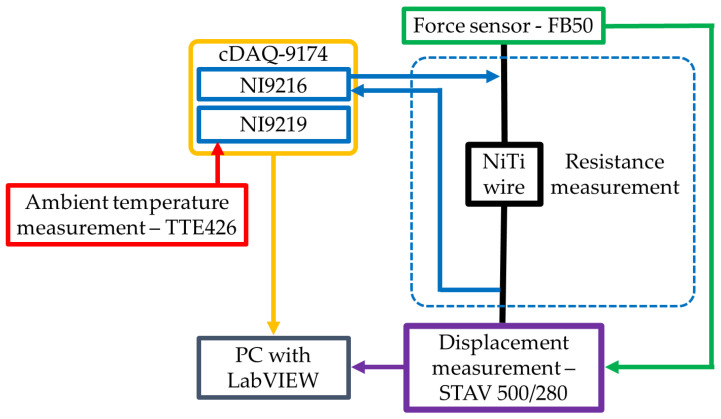
Schematic for the test stand.

The described test was realized by use of a developed test stand, the schema of which is presented in Figure 3. This test stand is based on the motorized vertical test stand, STAV 500/280 (AXIS Sp. z o.o., Gdańsk, Poland), the software of which was enabled to program the required stretching cycle. During the test, the displacement of the STAV 500/280 moving handle was measured at all times. This handle was also used to mount the force sensor FB50 (AXIS Sp. z o.o., Gdańsk, Poland), which was used during the test to measure the NiTi wire stretching force. FB50 is a dedicated STAV 500/280 force sensor with an accuracy of 0.05 N. During the test, one end of the fastened NiTi wire was fixed to the STAV 500/280, while the second end of the wire was attached to the FB50 force sensor that was mounted onto the moving handle of the STAV 500/280. The ring tongues of the prepared samples were not directly fixed to the STAV 500/280 and the FB50. Special clamps, made of PLA, were designed and manufactured by using the FDM method. The resistance of the NiTi wires was measured with a 24-bit analog module NI9216 (NI, Austin, TX, USA). This module was configured for a four-wire resistance measurement with a 1 mA stimulus current. According to the Joule-Lenz law, low stimulus current does not increase the temperature of a specimen, which is an important factor in the thermal behavior of NiTi alloy. The resistance measurement error of the NI9216, during the four-wire measurement at 25 °C, was 0.006 Ω. The second module NI9219 (NI, Austin, TX, USA) was used to measure the ambient temperature when using a TTE426 thermocouple. Both the NI9216 and NI9219 modules were connected with a cDAQ-9174 (NI, Austin, TX, USA) modular data acquisition system.

The measured data (i.e., the NiTi wire electrical resistance, the ambient temperature, the displacement of the moving handle, and the stretching force) were transmitted to a PC with a dedicated application installed. Communication between the cDAQ and the PC was realized via a USB cable that used a dedicated transmission protocol. The data obtained from the force sensor FB50 during the first stage was transferred to the STAV 500/280 test stand. Communication between the STAV 500/280 and the PC was created using a control unit via the USB virtual serial port. In both cases, the measured data from the cDAQ and STAV 500/280 were acquired at a frequency of 10 Hz.

The dedicated controlling software is written in the LabVIEW environment, for which there are three measurement streams that have to be controlled with different rates and protocols. The force and the displacement measurements were determined by STAV 500/280, which communicated with the computer via a USB serial protocol. The STAV 500/280 was in continuous operation, so it had to be handled at all times when the software was running, even when the making of measurements was inactive. The resistance measurements and the ambient temperature were found by NI9216 and NI9219, respectively. Both could be handled separately and could be switched on and off when the measurement procedure started or stopped. They communicated with the computer via a USB cable and a dedicated protocol that used a native LabVIEW library called DAQmx, which was employed. Each measurement was handled by its own separate parallel loop. The structure of the program consists of the main loop, called the Message Handling Loop, the two GUI loops (for handling user activities and displaying messages to the user), and three loops called “drivers” that handled the measurement devices described above. The base software structure is called a Queue Message Handler; this is because the main internal communication between the loops is based on queues.

The measurement data was stored in a TDMS file, which enabled storage of many measurement series in one file. The measurement series is called the group, the properties of which store information on, for example, the sample diameter, the length and the elongation, the date of measurement, the author etc. The synchronization of the data points was completed by using another programming structure called a Functional Global Variable. In fact, it is this type of function (known as the “save function”) that, when the measurement procedure starts a new file or a new group, creates data in an existing file. Each driver calls a save function with input parameters, which consists of measurement data alongside a timestamp. The latter are compared to each other and, if the difference is less than the maximum delay, the data is stored in the file as one measurement point that consists of all measurement values. At the end, the measurement file was closed. This solution enabled us to run short measurement series similar to those presented in this article, as well as long measurement series that are to be considered in future research.

## 3. Results

**Figure A1:**
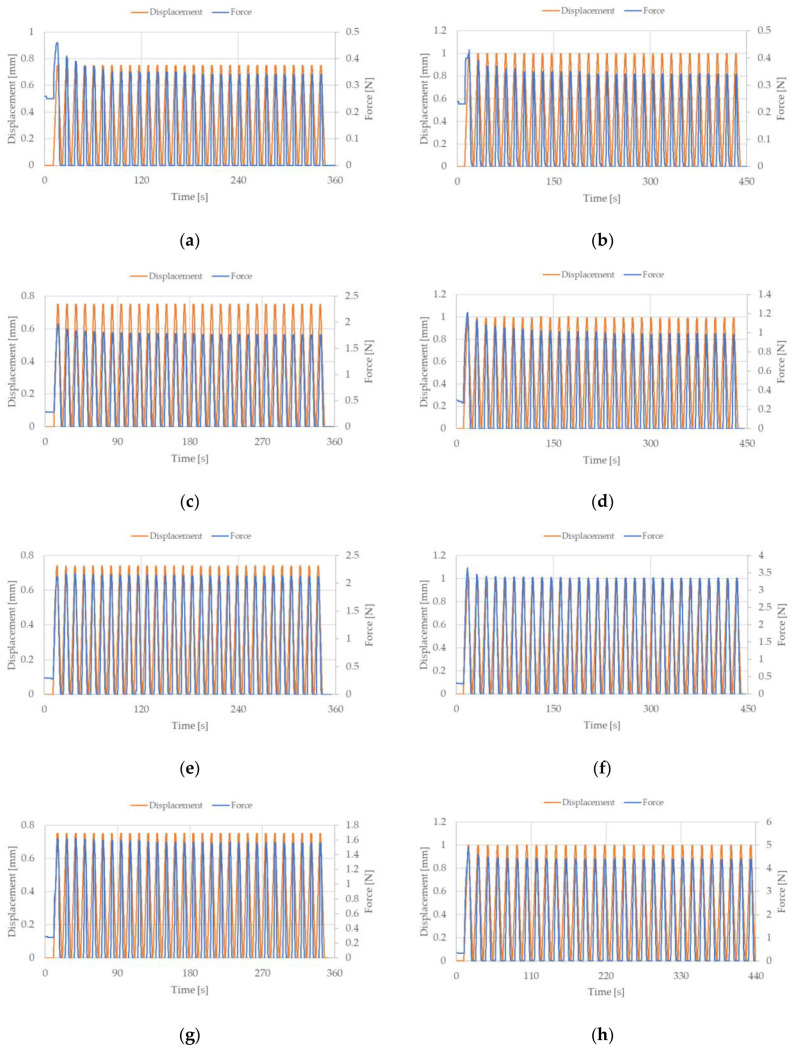
Force and deformation recorded during cyclic stretching of NiTi wires where stretching strain was 0.5%: (**a**) D = 100 µm, L = 150 mm; (**b**) D = 100 µm, L = 200 mm; (**c**) D = 150 µm, L = 150 mm; (**d**) D = 150 µm, L = 198 mm; (**e**) D = 200 µm, L = 148 mm; (**f**) D = 200 µm, L = 200 mm; (**g**) D = 250 µm, L = 149 mm; (**h**) D = 250 µm, L = 200 mm.

**Figure A2:**
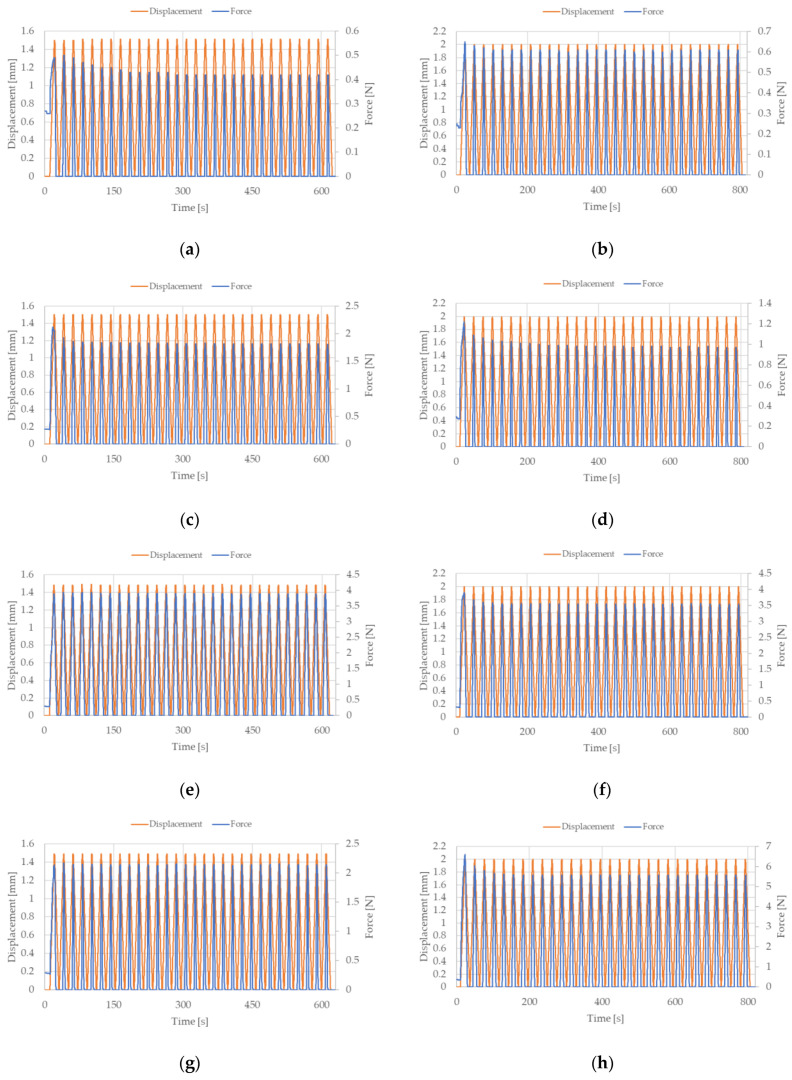
Force and deformation recorded during cyclic stretching of NiTi wires where stretching strain was 1%: (**a**) D = 100 µm, L = 150 mm; (**b**) D = 100 µm, L = 200 mm; (**c**) D = 150 µm, L = 150 mm; (**d**) D = 150 µm, L = 198 mm; (**e**) D = 200 µm, L = 148 mm; (**f**) D = 200 µm, L = 200 mm; (**g**) D = 250 µm, L = 149 mm; (**h**) D = 250 µm, L = 200 mm.

During the tests, electrical resistance, force, and deformation of NiTi wires were measured. The ambient temperature during the test was observed to be between 22 and 24 °C, which did not change the resistance of the tested samples to any noticeable extent. The force registered by the FB50 sensor and the deformation registered by STAV 500/280, during the cyclic stretching of the NiTi wires, are displayed in the graphs of Figure A1 and Figure A2 for the case of a stretching strain at 0.5% and 1%, respectively. The measured displacements indicate that the assumed stretching cycles did not have any distortions for all the analyzed configurations. At time t = 0, the force was equal to the applied preload, which is similar in all the examined cases (ca. 0.3 N). The maximum force peak, during each stretching cycle, was comparable in all the tests and it depended on the sample tested. The highest force peak was observed during the first stretching cycle in all the analyzed samples. In the case of NiTi wires with a diameter of 100 µm, the force peaks in the next stretching cycles gradually reduced, until they finally stabilized at a constant level after a few stretching cycles. This force peak stabilization occurred at a faster rate for NiTi wires with larger diameters. In fact, the larger the diameter of the NiTi wire, the faster the force peak stabilization. The measured force, following the first stretching cycle, dropped to 0 in all the examined cases. This indicates that the length of the NiTi wire changed (the tested samples were temporarily lengthened, due to martensite reorientation).

**Figure 4:**
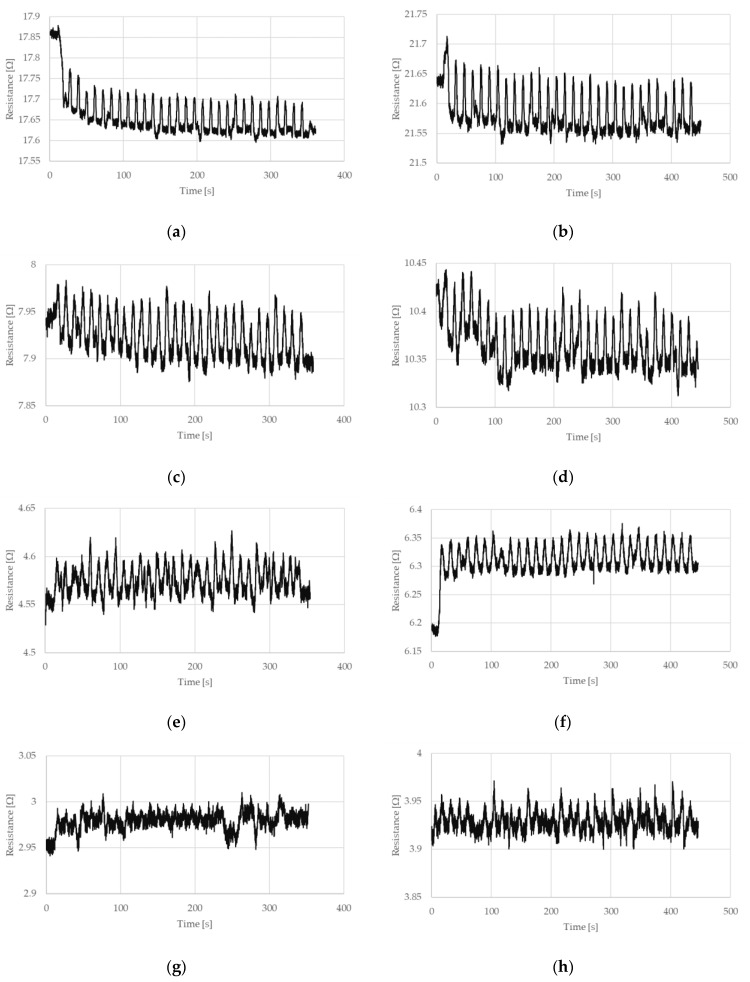
Resistance of NiTi wires recorded during cyclic stretching, in which the stretching strain is 0.5% and (**a**) the diameter, D = 100 µm and the length, L = 150 mm, (**b**) D = 100 µm and L = 200 mm, (**c**) D = 150 µm and L = 150 mm, (**d**) D = 150 µm and L = 198 mm, (**e**) D = 200 µm and L = 148 mm, (**f**) D = 200 µm and L = 200 mm, (**g**) D = 250 µm and L = 149 mm, and (**h**) D = 250 µm and L = 200 mm.

**Figure 5:**
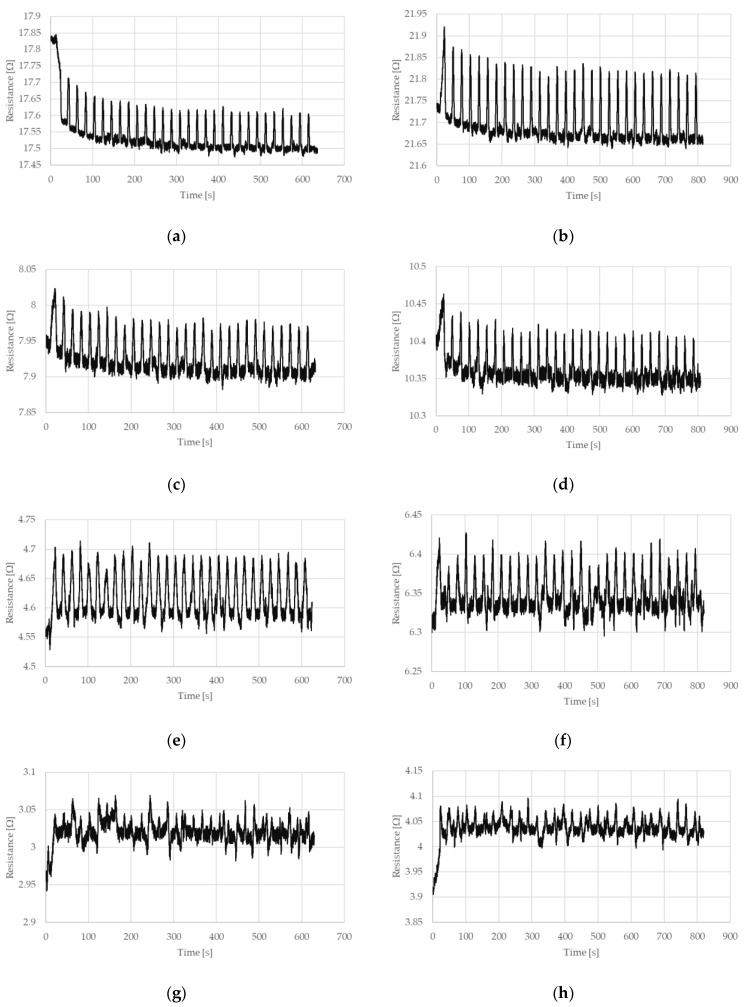
Resistance of NiTi wires recorded during cyclic stretching, in which the stretching strain is 1% and (**a**) the diameter, D = 100 µm and the length, L = 150 mm, (**b**) D = 100 µm and L = 200 mm, (**c**) D = 150 µm and L = 150 mm, (**d**) D = 150 µm and L = 198 mm, (**e**) D = 200 µm and L = 148 mm, (**f**) D = 200 µm and L = 200 mm, (**g**) D = 250 µm and L = 149 mm, and (**h**) D = 250 µm and L = 200 mm.

The measured electrical resistances of the tested NiTi wires are presented in Figure 4 and Figure 5 for the case of a stretching strain at 0.5% and 1%, respectively. It can be observed that the resistance of NiTi wires is related to its deformation. If this deformation increases, the electrical resistance also increases. The largest change in electrical resistance is seen for the first stretching cycle; this also occurred for the measured stretching force. Interesting parts of the presented charts relate to the time when the test stand movement was stopped. As can be seen in Figure 4 and Figure 5, the maximum and minimum peaks for electrical resistance differ, which depended on the number of performed stretching cycles. This is especially visible when the wires have the diameter of 100 or 150 µm.

## 4. Discussion

**Figure 6:**
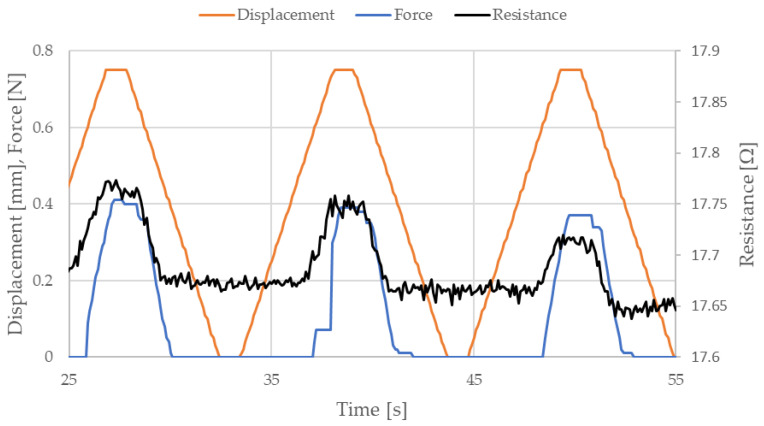
Selected parts of the measured data for cyclic stretching of a NiTi wire with diameter 100 µm and length 150 mm, where the strain is 0.5%.

Measured data related to NiTi wire electrical resistance can be divided into three sections; this will be discussed using the example data presented in Figure 6.

The data presented in Figure 6 displays almost three stretching cycles of NiTi wire, which has a diameter 100 µm, length 150 mm, and a stretching strain of 0.5%. As mentioned previously, the electrical resistance of NiTi wire depends on its displacement. When the test stand movement was stopped, in a position where this displacement was at a maximum (in the case of this sample, this was at 0.75 mm), the electrical resistance of the tested sample remained similar. Therefore, the first section responds to the part of the stretching cycle in which displacement is at a maximum and there is no movement. The second section responds to the portion of the cycle in which the displacement is at a minimum and there is again no movement. The third section relates to the stretching force. As seen in Figure 6, the stretching force was equal to 0 when the displacement was greater than 0 (when the moving handle had not yet returned to its initial position). This situation occurs because the distance between the moving and the fixed fastener is set up based on the free length of the NiTi wire and the applied preload. When the test begins, the sample length is increased due to martensite reorientation. Due to this, the length of the sample is slightly larger than the distance between the moveable and the fixed fasteners. This explains why the stretching force is equal to 0 before the system returns to its initial position. However, the conducted research indicated that the electrical resistance of NiTi wires was similar in the second and third sections [41]. In the next part of this article, electrical resistance of NiTi wires was obtained based on electrical resistance in the second section.

**Figure 7:**
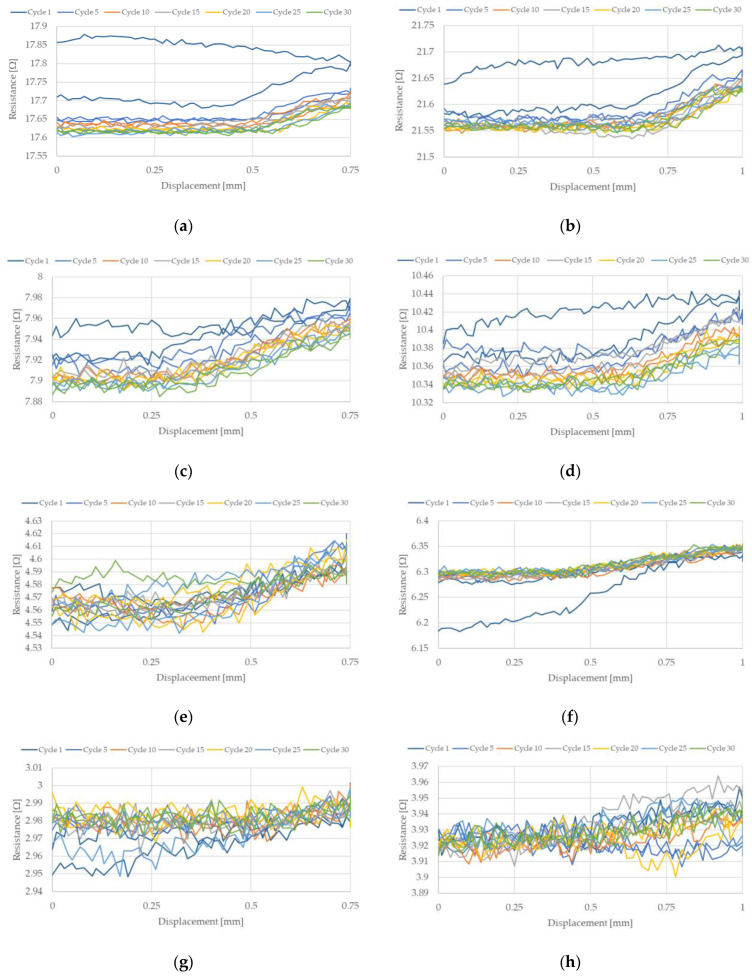
NiTi wire resistance change during selected stretching cycles where strain was 0.5%: (**a**) D = 100 µm, L = 150 mm; (**b**) D = 100 µm, L = 200 mm; (**c**) D = 150 µm, L = 150 mm; (**d**) D = 150 µm, L = 198 mm; (**e**) D = 200 µm, L = 148 mm; (**f**) D = 200 µm, L = 200 mm; (**g**) D = 250 µm, L = 149 mm; (**h**) D = 250 µm, L = 200 mm.

**Figure 8:**
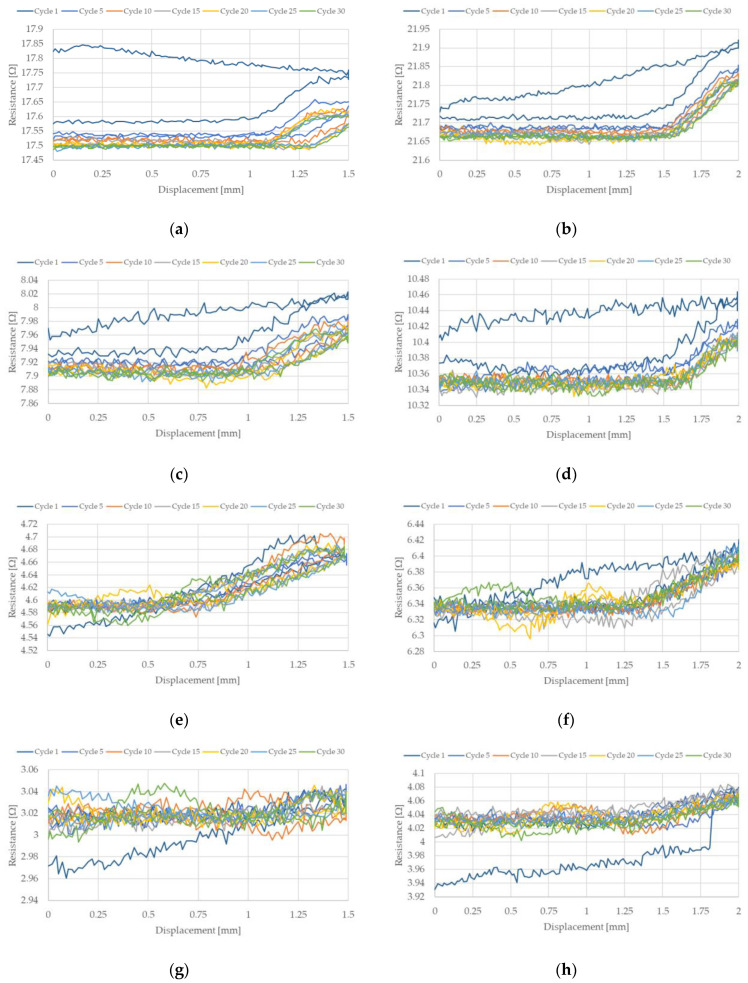
NiTi wire resistance change during selected stretching cycles where strain was 1%: (**a**) D = 100 µm, L = 150 mm; (**b**) D = 100 µm, L = 200 mm; (**c**) D = 150 µm, L = 150 mm; (**d**) D = 150 µm, L = 198 mm; (**e**) D = 200 µm, L = 148 mm; (**f**) D = 200 µm, L = 200 mm; (**g**) D = 250 µm, L = 149 mm; (**h**) D = 250 µm, L = 200 mm.

**Figure 9:**
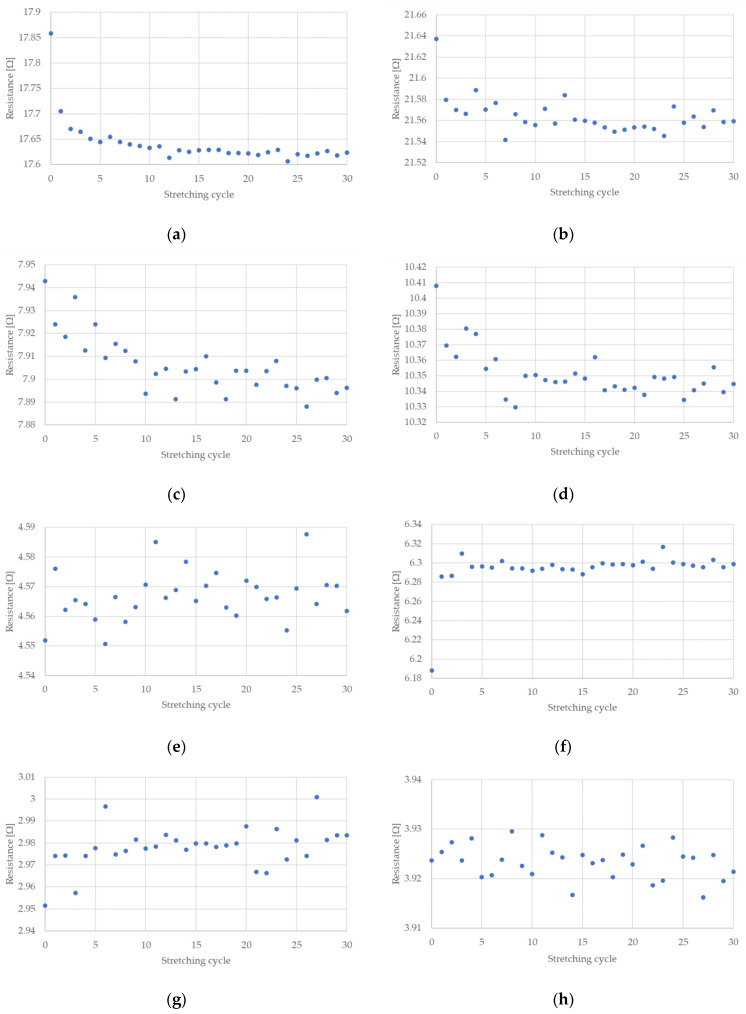
Average resistance of NiTi wires after each stretching cycle where elongation was 0.5%: (**a**) D = 100 µm, L = 150 mm; (**b**) D = 100 µm, L = 200 mm; (**c**) D = 150 µm, L = 150 mm; (**d**) D = 150 µm, L = 198 mm; (**e**) D = 200 µm, L = 148 mm; (**f**) D = 200 µm, L = 200 mm; (**g**) D = 250 µm, L = 149 mm; (**h**) D = 250 µm, L = 200 mm.

**Figure 10:**
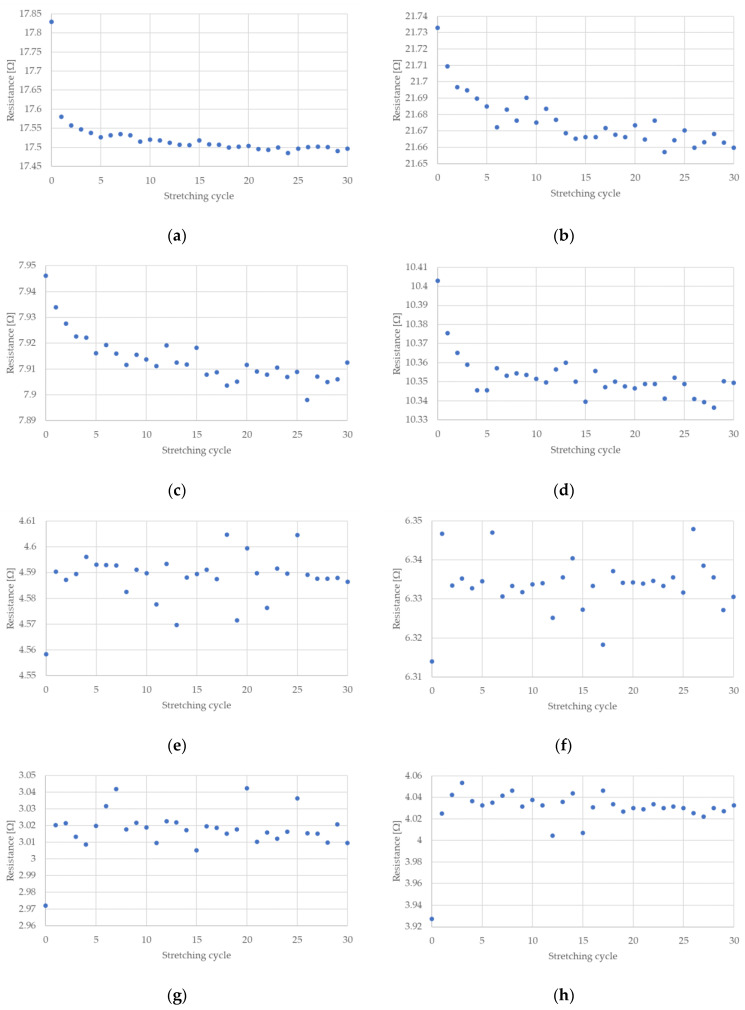
Average resistance of NiTi wires after each stretching cycle where elongation was 1%: (**a**) D = 100 µm, L = 150 mm; (**b**) D = 100 µm, L = 200 mm; (**c**) D = 150 µm, L = 150 mm; (**d**) D = 150 µm, L = 198 mm; (**e**) D = 200 µm, L = 148 mm; (**f**) D = 200 µm, L = 200 mm; (**g**) D = 250 µm, L = 149 mm; (**h**) D = 250 µm, L = 200 mm.

The change in NiTi resistance during cyclic stretching is related to the occurrence of a hysteresis loop. Such loops, corresponding to the resistance of the NiTi wires, for selected stretching cycles, are presented in Figure 7 and Figure 8. These hysteresis loops show the difference between NiTi wire electrical resistance when comparing the beginning and the end of selected stretching cycles. As seen in Figure 7 and Figure 8, the largest change in electrical resistance occurred during the first stretching cycle. The average value of electrical resistance for the tested wires in the second section, during all the stretching cycles, are presented in Figure 9 and Figure 10. As mentioned earlier, the largest change in NiTi wire electrical resistance is observed during the first stretching cycle. Independent from the applied stretching, the elongation of the tested samples can be divided into two groups. In the first group (for wires with diameters 100 µm and 150 µm), resistance, following the first stretching cycles, decreased. In the second group (for wires with diameters 200 µm and 250 µm), resistance after the initial stretching cycles increased.

For the first group, it can be seen that the resistance in the second section descended in each stretching cycle. This phenomenon is not observed in the samples from group two. It is noteworthy that the change in resistance for samples with diameters 100 µm and 150 µm were small following each stretching cycle. Changes in the resistance for samples with a diameter of 100 µm seemed to be larger than those with a 150 µm diameter. The initial resistance of the wires with a diameter 100 µm was greater than for wires with a 150 µm diameter. Thus, the change in the resistance could be related to its initial resistance, since the smaller the diameter of the wire, the greater the resistance. For the tested samples with larger diameters (i.e., 200 µm and 250 µm) their initial electrical resistance changes were much smaller compared to the samples with smaller diameters. Therefore, changes in their electrical resistance during cyclic stretching probably also occurred, but they were too small to be measurable with the test stand.

Figure 9e–h and Figure 10e–h show that only one significant change in electrical resistance occurred during the test, for cases with diameters 200 µm and 250 µm. This resistance change arose during the first stretching cycle. For the remaining cycles, the resistance fluctuated around a certain value. For samples with diameters 100 µm and 150 µm, the change in electrical resistance was visible for almost all the stretching cycles. The hysteresis loops that are presented in Figure 7a–d and Figure 8a–d clearly indicate a decline in resistance at the end of each stretching cycle. This is also confirmed by the average resistance values presented in Figure 9a–d and Figure 10a–d. The discussed trends concern the electrical resistance changes that are clearly visible for both the lengths that are tested. However, in the cases of the longer samples, the results are more chaotic. This could be related with the elongation of the wire during cyclic stretching (especially during the first cycle). For the longer wires, the free length of the tested samples extends more and this could be the reason for the small amount of measurement fluctuations. The obtained results also show that the changes in the resistance of the wires were largest at the beginning and they decreased with each stretching cycle. Due to this, in future, it could be worth performing research that concerns cyclic stretching with smaller elongations. The latter would probably enable avoidance of such large changes in electrical resistance during the first stretching cycle. This would allow us to observe the decrease of electrical resistance in many more of the cycles. In the case of the elongation provided in this research, the change in the resistance after 30 cycles was much smaller compared to the initial cycles. However, a descending trend was observed. Therefore, in future, it could be worth performing tests with an elongation employed in this work (i.e., at 0.5%), but with many more stretching cycles.

The performed research shows that using the presented NiTi wires in effective sensors is a future possibility. For wires with smaller diameters (i.e., 100 µm and 150 µm), there is an opportunity to employ them within cyclic working sensors, because the difference in electrical resistance is visible for almost all the stretching cycles. For NiTi wires with larger diameters (i.e., 200 µm and 250 µm), their use in cyclic sensors is practically impossible, since resistance change is unclear compared to wires with smaller diameters. However, the conducted research shows that they are highly sensitive to deformation events and, thus, they could be used to monitor the maximum deformations of larger structures (e.g., composites). The great advantage of this type of application is that it is useable both online and offline. On exceeding the allowable deformation of the structure, monitoring by NiTi-based sensors could still be detectable after the deformation, due to change in the NiTi wires (and the corresponding change in resistance) relating to martensite reorientation. This change will be permanent until the martensite-austenite-martensite phase transformation is caused by heating. After such a heat-phase transformation, the NiTi-based sensor would return to its initial indications and become reusable again. The performed research showed that NiTi-based sensors could be used in the future in self-sensing applications and in structural health monitoring.

## 5. Conclusions

The performed research showed that NiTi wires are very sensitive to deformation. During deformation of NiTi wires, a martensite reorientation occurs. This transformation affects the properties of the material, such as electrical resistance. Due to martensite reorientation, the length of the tested wires also changed (especially for the first stretching cycles). Electrical resistance of all the tested NiTi wires was significantly changed during the first stretching cycle. Change in electrical resistance as a function of displacement of the wire creates hysteresis loops. Resistance changes in the NiTi wires with smaller diameters (i.e., 100 µm and 150 µm) was slightly different compared to wires with larger diameters (i.e., 200 µm and 250 µm). For smaller diameters resistance decreased after the first stretching cycle, while for larger diameters resistance increased after the same cycle. Moreover, smaller diameters (i.e., 100 µm and 150 µm) experienced a decrease in electrical resistance for almost all the stretching cycles, which was small, but measurable; this was most easily observed for the smallest diameter (i.e., 100 µm). The smaller diameter samples also had the highest initial resistance, since this is dependent on wire diameter. It is supposed that resistance change occurred in all tested wires, but larger diameters (i.e., 200 µm and 250 µm) had an initial resistance much smaller than for smaller diameters. Therefore, because change in electrical resistance during cyclic stretching is small with respect to the initial resistance, resistance changes for larger diameters were often too small to be measured with the developed test stand. The observed phenomenon can be used in the future to build sensors which are based on electrical resistance change in NiTi wires. Such sensors could be used in self-sensing applications or in structural health monitoring.

## Figures and Tables

**Table 1:** Diameters and lengths of the NiTi wires that were used in the tests.

NiTi Wire Diameter [µm]	NiTi Wire Free Length [mm]	Stretching Elongation [%]
100	150	0.5
	200	
	150	1
	200	
150	150	0.5
	198	
	150	1
	198	
200	148	0.5
	200	
	148	1
	200	
250	149	0.5
	200	
	149	1
	200	

## Data Availability

Data is contained within the article.

## Appendix A

The force registered by the FB50 sensor and the deformation registered by the STAV 500/280, during the cyclic stretching of the NiTi wires, are presented in Figure A1 and Figure A2 for stretching strains 0.5% and 1%, respectively.

## References

[1] Duda S., Gąsiorek D., Gembalczyk G., Kciuk S., Mężyk A. (2016). Mechatronic device for locomotor training. Acta Mech. Autom..

[2] Herbin P., Pajor M. (2021). Human-robot cooperative control system based on serial elastic actuator bowden cable drive in ExoArm 7-DOF upper extremity exoskeleton. Mech. Mach. Theory.

[3] Dunaj P., Dolata M., Powalka B., Pawelko P., Berczynski S. (2021). Design of an Ultra-Light Portable Machine Tool. IEEE Access.

[4] Duda S., Dudek O., Gembalczyk G., Machoczek T. (2020). Developing a test site for testing the suspension of vehicles with omnidirectional wheels. Vib. Phys. Syst..

[5] Kciuk S., Krzystała E., Mężyk A., Szmidt P. (2022). The Application of Microelectromechanical Systems (MEMS) Accelerometers to the Assessment of Blast Threat to Armored Vehicle Crew. Sensors.

[6] Duda S., Gembalczyk G., Machoczek T., Szyszka P. (2021). Use of 3D Printing in Designing Sensor Overlays Used to Determine the Foot Pressure Distribution on the Ground. Adv. Intell. Syst. Comput..

[7] Miądlicki K., Saków M., Trojanowska J., Ciszak O., Machado J., Pavlenko I. (2019). LiDAR based system for tracking loader crane operator. Advances in Manufacturing II.

[8] Duda S., Dudek O., Gembalczyk G., Machoczek T. (2021). Determination of the Kinematic Excitation Originating from the Irregular Envelope of an Omnidirectional Wheel. Sensors.

[9] Panasiuk K., Dudzik K. (2022). Determining the Stages of Deformation and Destruction of Composite Materials in a Static Tensile Test by Acoustic Emission. Materials.

[10] Panasiuk K., Dudzik K., Hajdukiewicz G. (2021). Acoustic emission as a method for analyzing changes and detecting damage in composite materials during loading. Arch. Acoust..

[11] Katunin A., Pawlak S., Wronkowicz A. (2018). Evaluation of Structural Degradation of Polymeric Composites Subjected to Self-Heating by the Thermal Diffusivity Analysis. Arab. J. Sci. Eng..

[12] Wronkowicz-Katunin A., Katunin A., Nagode M., Klemenc J. (2021). Classification of Cracks in Composite Structures Subjected to Low-Velocity Impact Using Distribution-Based Segmentation and Wavelet Analysis of X-ray Tomograms. Sensors.

[13] Kowol P., Szczygieł M., Lo Sciuto G., Capizzi G. Modeling of Magnetorheological Fluids Relative Magnetic Permeability by Using a Neural Network Approach. Proceedings of the 2020 IEEE 20th Mediterranean Electrotechnical Conference (MELECON).

[14] Ivan A., Ardeleanu M., Lungu I., Gurgu V., Ionita M., Despa V., Catangiu A. (2015). A mixed piezoelectric and electromagnetic actuation device for dry and wet manipulation. Rom. Rev. Precis. Mech. Opt. Mechatron..

[15] Kciuk S., Kciuk M., Machoczek T., Klein W. (2019). Magnetorheological Suspension Based on Silicone Oil. Advances in Intelligent Systems and Computing.

[16] Kluszczyński K., Pilch Z. (2021). The Choice of the Optimal Number of Discs in an MR Clutch from the Viewpoint of Different Criteria and Constraints. Energies.

[17] Mohd Jani J., Leary M., Subic A., Gibson M.A. (2014). A review of shape memory alloy research, applications and opportunities. Mater. Des..

[18] Zhang J.-J., Yin Y.-H., Zhu J.-Y. (2013). Electrical Resistivity-Based Study of Self-Sensing Properties for Shape Memory Alloy-Actuated Artificial Muscle. Sensors.

[19] Sun L., Huang W.M. (2009). Nature of the multistage transformation in shape memory alloys upon heating. Met. Sci. Heat Treat..

[20] Mihálcz I. (2001). Fundamental Characteristics and Design Method for Nickel-Titanium Shape Memory Alloy. Period. Polytech. Mech. Eng..

[21] Lee S.H., Kim S.W. (2020). Self-sensing-based deflection control of carbon fibre-reinforced polymer (CFRP)-based shape memory alloy hybrid composite beams. Compos. Struct..

[22] Abavisani I., Rezaifar O., Kheyroddin A. (2021). Multifunctional properties of shape memory materials in civil engineering applications: A state-of-the-art review. J. Build. Eng..

[23] Ferčec J., Anžel I., Rudolf R. (2014). Stress dependent electrical resistivity of orthodontic wire from the shape memory alloy NiTi. Mater. Des..

[24] Prasad M.H. (2003). Development of shape memory alloy (SMA)-based actuator for remotely piloted vehicles (RPVs). Smart Mater. Struct. Syst..

[25] Amari M.D., Che Abdullah S. (2017). A Model for Hypersensitive Airflow Sensor Concept. J. Mech. Eng..

[26] Lan C.C., Lin C.M., Fan C.H. (2011). A self-sensing microgripper module with wide handling ranges. IEEE/ASME Trans. Mechatron..

[27] Mao C., Li H. (2005). SMA-based smart damper/displacement transducer. Smart Structures and Materials 2005: Sensors and Smart Structures Technologies for Civil, Mechanical, and Aerospace Systems.

[28] Pittaccio S., Garavaglia L., Ceriotti C., Passaretti F. (2015). Applications of Shape Memory Alloys for Neurology and Neuromuscular Rehabilitation. J. Funct. Biomater..

[29] Kciuk M., Kuchcik W., Pilch Z., Klein W. (2019). A novel SMA drive based on the Graham Clock escapement and resistance feedback. Sens. Actuators A Phys..

[30] Liu Z.-Q., Li H., Chen W.-L., Ou J.-P. (2005). Study on Strain-Sense Property of TiNi and TiNiCu Shape Memory Alloys. Adv. Struct. Eng..

[31] Rączka W., Konieczny J., Sibielak M. (2013). Laboratory Tests of Shape Memory Alloy Wires. Solid State Phenom..

[32] Šittner P., Sedlák P., Landa M., Novák V., Lukáš P. (2006). In situ experimental evidence on R-phase related deformation processes in activated NiTi wires. Mater. Sci. Eng. A.

[33] Uchil J., Mahesh K.K., Kumara K.G. (2002). Electrical resistivity and strain recovery studies on the effect of thermal cycling under constant stress on R-phase in NiTi shape memory alloy. Phys. B Condens. Matter.

[34] Wu X.D., Fan Y.Z., Wu J.S. (2000). A study on the variations of the electrical resistance for NiTi shape memory alloy wires during the thermo-mechanical loading. Mater. Des..

[35] Antonucci V., Faiella G., Giordano M., Mennella F., Nicolais L. (2007). Electrical resistivity study and characterization during NiTi phase transformations. Thermochim. Acta.

[36] Novák V., Šittner P., Dayananda G.N., Braz-Fernandes F.M., Mahesh K.K. (2008). Electric resistance variation of NiTi shape memory alloy wires in thermomechanical tests: Experiments and simulation. Mater. Sci. Eng. A.

[37] Furst S.J., Seelecke S. (2012). Modeling and experimental characterization of the stress, strain, and resistance of shape memory alloy actuator wires with controlled power input. J. Intell. Mater. Syst. Struct..

[38] Li H., Liu Z., Ou J. (2008). Experimental study of a simple reinforced concrete beam temporarily strengthened by SMA wires followed by permanent strengthening with CFRP plates. Eng. Struct..

[39] Wang T.-M., Shi Z.-Y., Liu D., Ma C., Zhang Z.-H. (2012). An Accurately Controlled Antagonistic Shape Memory Alloy Actuator with Self-Sensing. Sensors.

[40] Dudek O., Klein W., Gąsiorek D., Pawlak M. (2022). Additive Manufacturing of Smart Composite Structures Based on Flexinol Wires. Materials.

[41] Sławski S., Kciuk M., Klein W. (2021). Assessment of SMA Electrical Resistance Change during Cyclic Stretching with Small Elongation. Sensors.

